# Impact of smoking status and smoking index on outcomes in patients with acute basilar artery occlusion

**DOI:** 10.3389/fneur.2025.1623245

**Published:** 2025-10-31

**Authors:** Bin Han, Xu Tong, Raynald, Baixue Jia, Dapeng Mo, Feng Gao, Ning Ma, Xuan Sun, Zhongrong Miao

**Affiliations:** ^1^Shanxi Key Laboratory of Brain Disease Control, Department of Neurology, Shanxi Provincial People’s Hospital, Taiyuan, China; ^2^Department of Interventional Neuroradiology, Beijing Tiantan Hospital, Capital Medical University, Beijing, China; ^3^Department of Neurology, Beijing Tiantan Hospital, Capital Medical University, Beijing, China; ^4^China National Clinical Research Center for Neurological Diseases, Beijing Tiantan Hospital, Capital Medical University, Beijing, China; ^5^Dharmais Cancer Hospital, National Cancer Center, Jakarta, Indonesia

**Keywords:** smoking paradox, basilar artery occlusion, thrombectomy, smoking index, stroke

## Abstract

**Purpose:**

This study aims to investigate the relationship between smoking status, smoking index, and the outcomes of intravascular treatment for acute basilar artery occlusion within 24 h.

**Methods:**

We retrospectively analyzed all consecutive patients hospitalized with acute basilar artery occlusion who underwent endovascular treatment within 24 h from January 2012 to July 2018 at Beijing Tiantan Hospital. Smoking status was categorized as never smoking, current smoking, or previous smoking. The smoking index (SI) was calculated as the daily smoking count multiplied by the number of smoking years. The primary outcomes were a 90-day modified Rankin Scale score shift analysis and mortality at 90 days.

**Results:**

The overall study cohort comprised 59 never smokers, 58 former smokers, and 70 current smokers. No significant differences in primary outcomes were observed between smoking status and functional independence (OR, 1.611; 95% CI, 0.776–3.344) or death (OR, 0.461; 95% CI, 0.196–1.084). Multivariate analysis indicated that smoking status had limited relevance to functional independence (OR, 1.958; 95% CI, 0.781–4.907) and death (OR, 0.446; 95% CI, 0.169–1.178). The smoking index was independently associated with functional independence (OR, 1.095; 95% CI, 1.015–1.182) and death (OR, 0.844; 95% CI, 0.757–0.941). The smoking index demonstrated a dose–effect relationship with outcomes, being positively correlated with functional independence and negatively correlated with death.

**Conclusion:**

Smoking status does not appear to influence prognosis. However, the smoking index may be associated with improved functional independence and a reduced risk of death, demonstrating a dose-effect relationship.

## Introduction

Smoking is a major risk factor for ischemic stroke morbidity and mortality and is the leading cause of death worldwide. Smoking nearly doubles the risk of acute ischemic stroke (AIS). Despite its adverse health effects, some studies have identified a “smoking paradox,” suggesting that smokers may experience more favorable outcomes in AIS compared to nonsmokers (1). Additionally, a retrospective analysis study also showing that smoking was independently associated with lower inpatient mortality in acute ischemic stroke, potentially due to tobacco-induced changes in cerebrovascular vasoreactivity or residual confounding (2). Regarding endovascular treatments, Meseguer et al. (3) reported that intra-arterial rt-PA administration was more effective in achieving complete recanalization in both current and former smokers. Consistently, von Martia et al. (4) also found that smoking might predict excellent clinical outcomes after endovascular treatment (EVT), which is increasingly preferred for severe strokes with large artery occlusion. However, the reasons for this association are not fully understood, and conflicting evidence complicates the potential benefits of smoking on favorable outcomes after adjusting for confounders (5–7).

There is limited research on the relationship between smoking index and prognosis following EVT, specifically in patients with acute basilar artery occlusion (BAO). Therefore, this study aimed to retrospectively examine the smoking status and smoking index in patients with acute basilar artery occlusion who underwent endovascular treatment within 24 h.

## Methods

### Study design

We conducted a retrospective analysis of patients with BAO who underwent endovascular treatment (EVT) at our institution from January 2012 to July 2018. In accordance with current guidelines, intravenous thrombolysis (tissue plasminogen activator) was administered prior to EVT when indicated. Eligible patients received endovascular treatment within 24 h of symptom onset. Exclusion criteria included a modified Rankin Scale (mRS) score greater than 3 and incomplete information regarding cigarette smoking status, secondhand smoke exposure, and e-cigarette use.

### Study population

All participants provided informed consent. Clinical baseline variables were recorded, including age, gender, vascular risk factors, clinical characteristics, blood pressure, the National Institutes of Health Stroke Scale (NIHSS), laboratory tests, and stroke types classified according to the TOAST criteria from the ORG 10172 trial for acute stroke treatment. Additionally, anatomical and morphological characteristics of BAO, ASINT/SIR, and details of EVT were documented. Clinical outcomes and mortality within 90 days were prospectively collected. The time of BAO incidence was recorded based on patient or witness reports; if these were unavailable, the time of the last patient observation was used. For patients who experienced a sudden loss of consciousness following mild symptoms, the estimated time of clinical deterioration was used to approximate the onset of BAO. Functional outcomes at 90 days were assessed using the mRS. Follow-up for baseline variables was conducted by trained interviewers via standardized telephone interviews.

### Smoking definition

Smoking status was categorized into three groups: never smokers, current smokers, and former smokers. Current smokers were defined as individuals who smoked at least one cigarette per day and had been smoking continuously or cumulatively for at least 6 months prior to the stroke. Former smokers were those who had quit smoking for at least 6 months at the time of the survey. Individuals who had smoked less than 18 packs cumulatively and did not meet the criteria for current or former smoking were classified as never smokers. The smoking index (SI) was calculated as the product of daily cigarette consumption and the number of years of smoking (8).

### Clinical outcomes

In this study, the primary outcome measures were functional independence, favorable outcomes, and mortality at 90 days postoperatively. Functional independence was defined as a mRS score of ≤2. A favorable outcome was similarly defined as an mRS score of ≤2.

### Statistical analysis

Study data were collected using standardized forms, evaluated for completeness, and entered into SPSS for statistical analysis. Continuous variables were described using means (standard deviations) and/or medians (25th and 75th percentiles), while categorical variables were summarized using frequencies and/or proportions. Univariate analysis utilized independent sample *t*-tests or non-parametric tests (Mann–Whitney *U* test) to compare differences between means or medians, and Pearson chi-square tests or Fisher’s exact tests for comparing frequencies and proportions. Multivariate binary logistic regression was employed to determine independent predictors of 90-day outcomes, with adjusted odds ratios (OR) and 95% confidence intervals (CI) calculated. Variables with *p*-values ≤ 0.1 in univariate analysis or those showing more than a 10% variation in effect estimates were considered for inclusion in the multivariate model. Confounding factors were assessed, and collinearity was addressed. The smoking index, treated as a continuous variable, was analyzed using a spline smoothing function to evaluate its effect on thrombectomy outcomes in basilar artery occlusion (BAO). All tests were two-tailed, with statistical significance set at an alpha level of 0.05. Statistical analyses were conducted using the R software package (http://www-rprimt.org, R Foundation).

## Results

### Baseline characteristics of patients

From January 2012 to July 2018, a total of 187 patients with BAO ischemic stroke were enrolled in the study, all of whom received endovascular treatment within 24 h (Supplementary Figure 1). No patients were excluded. Based on smoking status, the cohort included 59 never smokers, 58 former smokers, and 70 current smokers. Patients were categorized according to their 90-day prognosis: 68 had a good prognosis (mRS 0–2), while 119 had a poor prognosis (mRS 3–6). Of the total, 149 patients survived and 38 patients died.

Baseline characteristics of patients with different outcomes are presented in Table 1. Demographic data did not show significant differences between the groups. However, a history of prior stroke was more prevalent in the survivor group compared to the non-survivor group (23.5% vs. 5.2%). Other risk factors, including hypertension, diabetes, and hypercholesterolemia, did not differ significantly between prognosis groups. Regarding clinical features, patients in the dependence group had a higher National Institutes of Health Stroke Scale (NIHSS) score at admission (29 vs. 10) compared to the survivors, while the non-survivor group had an even higher NIHSS score (34 vs. 17). Additionally, the dependence group had elevated white blood cell counts (11.38 × 10^9^/L vs. 10.16 × 10^9^/L) and creatinine levels (73.19 μmol/L vs. 65.18 μmol/L). No significant differences were observed in systolic blood pressure, blood glucose levels, or diffusion-weighted imaging (DWI)-ASPECTS scores. The proportion of patients with poor prognosis was higher among those receiving general anesthesia (86.6% vs. 64.7%), with a statistically significant difference noted in the 90-day modified Rankin Scale (mRS) scores (*p* < 0.001). Conversely, no significant difference in prognosis was observed between survivors and non-survivors based on the type of anesthesia. Additionally, the use of stents (79.8% vs. 63.2%) and balloon dilation (73.9% vs. 57.4%) was more common in the poor prognosis group, with a significant difference in the 90-day mRS scores. However, no significant differences were found between survivors and non-survivors with respect to these interventions.

**Table 1:** Baseline characteristics of smoking and nonsmoking patients with different outcomes.

Variable names	Overall (*n* = 187)	Independence (mRS 0–2, *n* = 68)	Dependence (mRS 3–6, *n* = 119)	*p*-value^*^	Death (*n* = 38)	Survival (*n* = 149)	*p-*value^a^
Demographic data							
Age, mean (SD), years	60 (10)	59 (11)	60 (10)	0.582	59 (10)	60 (10)	0.663
Male sex	157(84.0)	59 (86.8)	98 (82.4)	0.429	30 (78.9)	127 (85.2)	0.346
Vascular risk factors							
Hypertension	133 (71.1)	49 (72.0)	84 (70.6)	0.831	27 (71.0)	106 (71.1)	0.991
Diabetes mellitus	51 (27.3)	16 (23.5)	35 (29.4)	0.385	15 (39.4)	36 (24.1)	0.059
Hypercholesterolemia	30 (16.0)	13 (19.1)	17 (14.3)	0.386	6 (15.8)	24 (16.1)	0.962
Prior stroke	37 (19.8)	13 (19.1)	24 (20.2)	0.862	2 (5.2)	35 (23.5)	0.012
Clinical characteristics							
SBP, mean (SD), mmHg	160 (25)	162 (25)	159 (26)	0.417	160 (31)	160 (24)	0.981
NIHSS score, median (IQR)	22 (10–34)	10 (4–20)	29 (16–35)	<0.001	34 (27–35)	17 (8–31)	<0.001
WBCs, mean (SD), ×10^9^/L	10.94 (3.86)	10.16 (3.84)	11.38 (3.81)	0.039	11.73 (4.16)	10.74 (3.16)	0.167
Blood glucose, mean (SD), mmol/L	8.9 (3.6)	8.9 (3.2)	8.9 (3.7)	0.929	9.70 (4.7)	8.7 (3.2)	0.152
Creatinine, mean (SD), umol/L	70.26 (21.57)	65.18 (16.01)	73.19 (23.76)	0.015	68.88 (22.26)	75.80 (22.17)	0.081
pc-ASPECTS on DWI, median (IQR)	6 (5–8)	7 (5–8)	6 (5–8)	0.230	6 (5–8)	7 (5–8)	0.626
Occlusion site				0.057			0.544
Proximal BA (including intracranial VA)	104 (55.6)	45 (66.2)	59 (49.6)		19 (50.0)	85 (57.0)	
Middle BA	54 (28.9)	13 (19.1)	41 (34.5)		11 (28.9)	43 (28.9)	
Distal BA	29 (15.5)	10 (14.7)	19 (16.0)		8 (21.1)	21 (14.1)	
Underlying ICAS	117 (62.6)	43 (63.2)	74 (62.2)	0.886	20 (52.6)	97 (65.1)	0.156
Stroke subtype by TOAST criteria				0.813			0.791
Large artery arteriosclerosis	157 (84.0)	54 (79.4)	97 (81.5)		31 (81.6)	120 (80.5)	
Cardioembolic	29 (15.5)	12 (17.6)	17 (14.3)		5 (13.2)	24 (16.1)	
Other or unknown etiology	7 (3.7)	2 (2.9)	5 (4.2)		2 (5.2)	5 (3.4)	
Procedural features							
Prior use of intravenous tPA	36 (19.3)	15 (22.1)	21 (17.6)	0.462	6 (15.8)	30 (20.1)	0.544
General anaesthesia	147 (78.6)	44 (64.7)	103 (86.6)	<0.001	34 (89.5)	113 (75.8)	0.067
Use of stent retriever	138 (73.8)	43 (63.2)	95 (79.8)	0.013	32 (84.2)	106 (71.1)	0.102
No. of passes, median (IQR)	1 (1–2)	1 (1–2)	2 (1–2)	0.172	2 (1–3)	1 (1–2)	0.012
Intra-arterial tPA or Urokinase	43 (23.0)	15 (22.0)	28 (23.5)	0.818	13 (34.2)	30 (20.1)	0.066
Angioplasty(Balloon or Stenting)	127 (67.9)	39 (57.4)	88 (73.9)	0.019	27 (71.1)	100 (67.1)	0.642
Onset to puncture time, median (IQR), hours	7 (5–9)	6 (4.5–8.88)	7 (5–10)	0.250	8 (4.88–10.5)	7 (5–9)	0.775
Astin collaterals	2 (1–2)	2 (1–2)	2 (1–2)	0.006	1 (1–2)	2 (1–2)	0.077

Values are numbers with percentages in parentheses, unless indicated otherwise.

^*^Independence vs dependence.

a Death vs survival.

BA, basilar artery; DWI, diffusion weighted imaging; ICAS, intracranial atherosclerotic stenosis; IQR, interquartile range; LDL, low density lipoprotein; mRS, modified Rankin scale; NIHSS, National Institutes of Health Stroke Scale; pc-ASPECTS, posterior circulation acute stroke prognosis early MRI score; SBP, systolic blood pressure; SD, standard deviation; ASITN/SIR, American Society of Interventional and Therapeutic Neuroradiology/Society of Interventional Radiology; TOAST, trial of org 10,172 in acute stroke treatment; tPA, tissue plasminogen activator; VA, vertebral artery; WBC, white blood cell. Angioplasty = Balloon or Stenting in Intravascular mechanical thrombectomy.

### Smoking status and outcome

Endovascular treatment outcomes were analyzed based on smoking history, categorizing patients into three groups: (1) never smokers, (2) former smokers, and (3) current smokers. No significant differences were found in 90-day modified Rankin Scale (mRS) scores or survival rates among these groups. Univariate analysis revealed no significant association between smoking status and functional independence [odds ratio (OR), 1.611; 95% confidence interval (CI), 0.776–3.344] or mortality (OR, 0.461; 95% CI, 0.196–1.084), with *p* > 0.1. To account for potential confounding factors and collinearity, a multivariate logistic regression analysis was performed, including variables with significant univariate associations (*p* < 0.1) and those showing more than a 10% change in effect estimates. Factors considered included preoperative NIHSS score, preoperative white blood cell count, preoperative serum creatinine level, lesion site, DSA-based ASIPN/SIR collateral grading, general anesthesia, stent thrombectomy, and angioplasty (balloon dilation or stent implantation). The analysis showed that smoking status was not significantly associated with a good prognosis (mRS 0–2) or with mortality after adjusting for these factors. The presence of general anesthesia and the number of stents also did not significantly correlate with mortality.

### Smoking index and outcome

**Figure 1:**
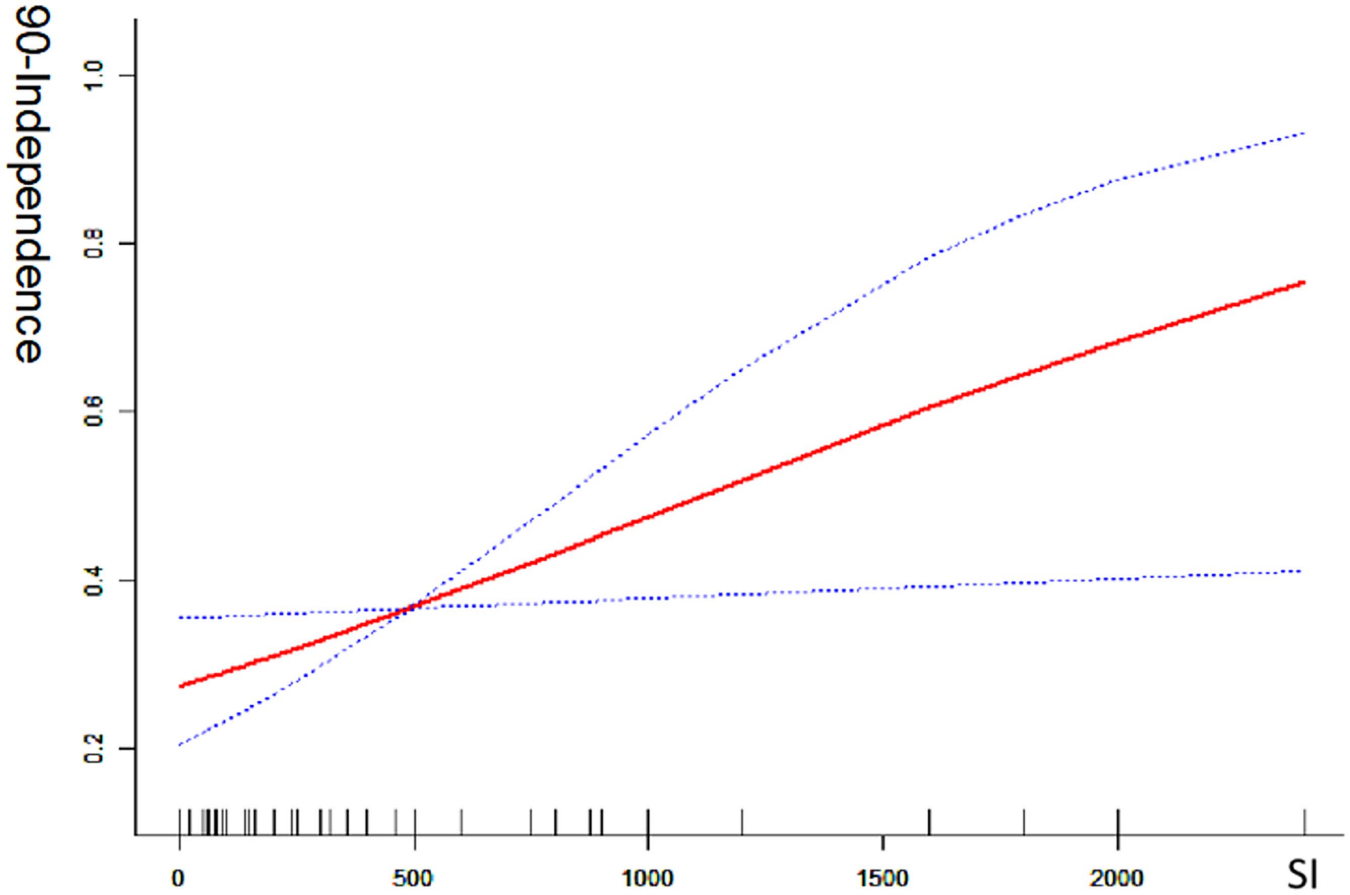
Good 90-day functional outcome with smoking index; 90-independence indicates modified Rankin scale (mRS 0–2) at 90 day ratio; SI indicates smoking index; smoking index (SI) = daily smoking count × number of smoking years.

**Figure 2:**
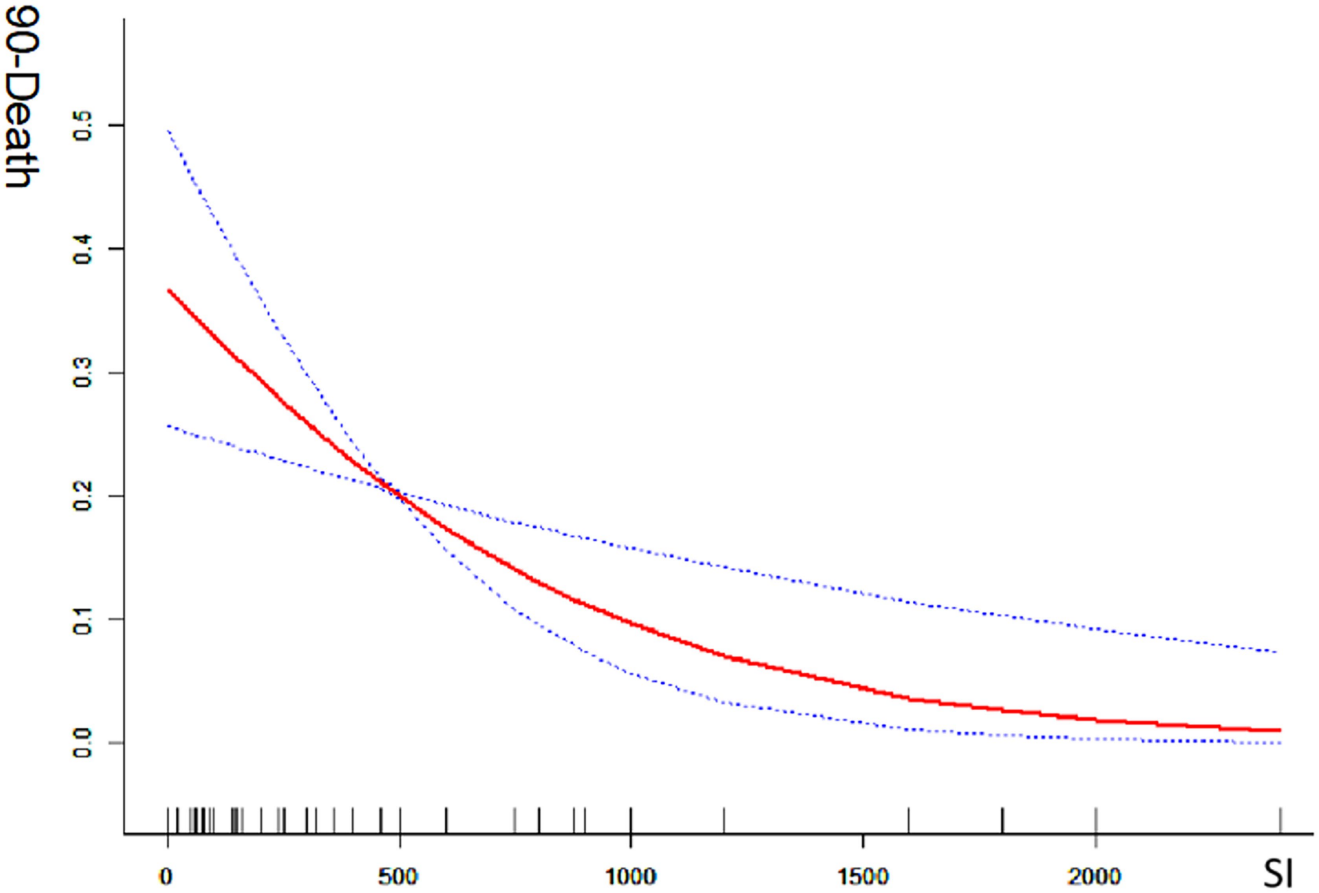
Mortality at 90-days with smoking index; 90-death indicates 90-day mortality ratio; SI indicates smoking index; smoking index (SI) = daily smoking count × number of smoking years.

Univariate analysis revealed that the smoking index was significantly associated with a good prognosis [odds ratio (OR) = 1.071; 95% confidence interval (CI) = 1.011–1.134; *p* < 0.05] and with mortality (OR = 0.862; 95% CI = 0.781–0.951; *p* < 0.05). In multivariate analysis, which adjusted for preoperative NIHSS score, white blood cell count, serum creatinine level, lesion site, DSA-based ASIPN/SIR collateral grading, general anesthesia, and angioplasty (excluding stent thrombectomy), the smoking index remained significantly associated with a good prognosis (OR = 1.019; 95% CI = 1.015–1.182; *p* < 0.05). This indicates a positive correlation between the smoking index and the likelihood of a good prognosis (mRS 0–2). Conversely, when adjusted for the same factors, the smoking index was also significantly correlated with reduced mortality (OR = 0.844; 95% CI = 0.757–0.941; *p* < 0.05), indicating a negative correlation with death. The smoking index, treated as a continuous variable, was further analyzed using spline smoothing. The results indicated a linear relationship between the smoking index and good prognosis, with higher smoking index values associated with a higher probability of a good prognosis (Figure 1). Conversely, there was an approximately linear negative correlation between the smoking index and mortality, with higher smoking index values associated with a lower probability of death (Figure 2).

## Discussion

Our study demonstrates that a higher smoking index is associated with a better prognosis and lower in-hospital mortality. This association persists even after extensive adjustments for various covariates, extending the “smoking paradox” observed in other conditions. However, the available data is limited, and the underlying biological mechanisms explaining the observed benefits in smokers are not yet fully understood.

Many studies propose that the “smoking paradox” may be attributed to confounding factors, including the lower risk of acute ischemic stroke in younger and current smokers (3, 9–11). Additionally, male gender and older age are associated with poorer clinical outcomes (12, 13). Despite controlling for these confounding variables, some studies have found that in-hospital mortality remains lower in smokers compared to non-smokers (1, 2, 4). In our study, baseline characteristics, including age and risk factors, showed no significant differences between the groups, suggesting that the paradox cannot be fully explained or eliminated.

Our findings indicate that a higher smoking index is associated with better prognosis and lower in-hospital mortality. This observation may be attributed to smokers potentially having “more favorable” atherosclerotic profiles and, consequently, better outcomes compared to non-smokers (14). One possible explanation is that smoking induces a hypercoagulable state, which might enhance the effectiveness of thrombolytic therapy. This hypercoagulability is associated with increased endothelial dysfunction, elevated platelet activation and aggregation, higher thrombin generation, and elevated circulating fibrinogen levels, all of which can impair endogenous fibrinolysis (15–17). Conversely, non-smokers may experience more frequent occlusions due to the rupture or ulceration of atherosclerotic plaques, which form platelet-rich thrombi (18). These thrombi might respond more effectively to intravascular mechanical thrombectomy, potentially improving vascular recanalization rates and cerebral perfusion. As a result, smokers might achieve better prognoses and lower mortality rates following treatment. However, the current study lacks supporting evidence and further investigation is warranted to validate these findings.

A dose–response relationship has been established between the number of cigarettes smoked per day and circulating fibrinogen levels (19, 20). Our study indicates that a higher smoking index is associated with a better prognosis and lower mortality. This suggests that a certain level of smoking may confer a protective effect on the vessel wall, potentially enhancing the response to both spontaneous and therapeutic thrombolysis. Furthermore, smoking is associated with persistently elevated levels of carbon monoxide in the plasma, which may induce adaptive cellular responses akin to those seen with ischemic preconditioning. These responses could potentially make smokers more resilient to ischemic damage, thereby offering better preconditioning effects during ischemic episodes (21, 22).

We acknowledge several limitations in this study. First, the modest sample size, particularly in subgroup analyses, may reduce statistical power. Nevertheless, the consistency of results across both univariate and multivariate models, together with their concordance with prior studies, supports the reliability of our findings. While we consider this study an important contribution to the field, larger multicenter datasets are required for confirmation. Second, our mechanistic explanations remain speculative and are primarily based on prior literature. Future studies incorporating laboratory biomarkers (e.g., fibrinogen levels, platelet function assays) and imaging data (e.g., thrombus composition, collateral circulation quality) are needed to provide more direct evidence linking the smoking index to stroke outcomes. Third, as an observational study, it is subject to treatment biases that cannot be fully eliminated by multivariate adjustment. Fourth, we did not account for the use of other tobacco products, which may have introduced confounding if participants used these concurrently. Fifth, unmeasured factors such as rehabilitation patterns, socioeconomic status, and medical complications could also have influenced the outcomes. Finally, changes in smoking habits, including post-stroke cessation, were not assessed, which may have affected the results observed at 3 months. Despite these limitations, a key strength of our study lies in the application of advanced thrombectomy stents in real-world cases of basilar artery occlusion (BAO). By carefully adjusting for potential confounders, we sought to minimize the risk of false-positive findings. Further multicenter studies with larger sample sizes are warranted to validate these results.

## Conclusion

Our data suggest that smoking status itself does not significantly affect prognosis. However, the smoking index appears to be associated with improved functional independence and a lower risk of mortality, showing a dose–effect relationship. Further prospective, population-based studies are required to investigate these findings more comprehensively.

## Data Availability

The original contributions presented in the study are included in the article/Supplementary material, further inquiries can be directed to the corresponding authors.
