# Supplementary figures and images for: Impact of smoking status and smoking index on outcomes in patients with acute basilar artery occlusion
Source: Front Neurol. 2025 Oct 31;16:1623245. doi: 10.3389/fneur.2025.1623245 (PMC12615231; doi:10.3389/fneur.2025.1623245)

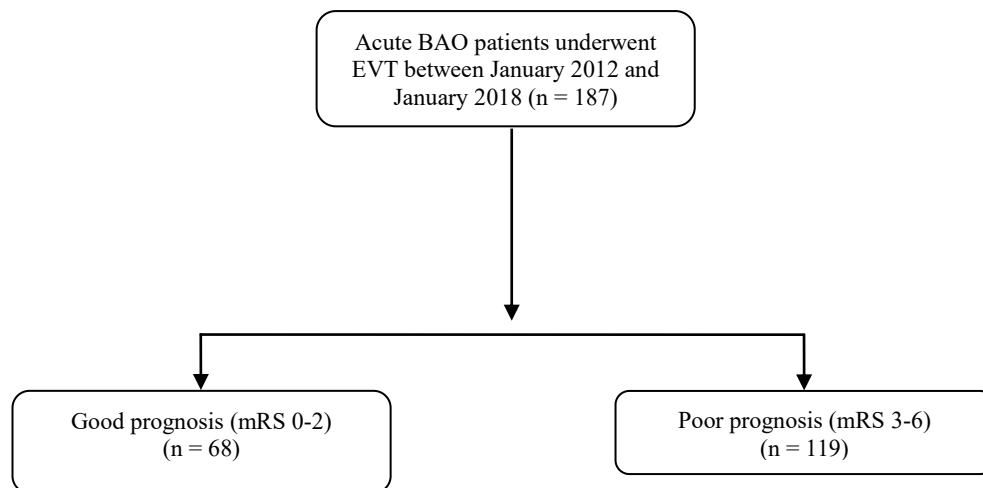

**Supplementary figure.**

Supplement: Supplementary file 1 [file Data_Sheet_1.pdf]
